# Editorial on the Special Issue “Novel Gels for Food Product Development”

**DOI:** 10.3390/gels9070520

**Published:** 2023-06-27

**Authors:** Anna Florowska, Tomasz Florowski, Osvaldo H. Campanella

**Affiliations:** 1 Department of Food Technology and Assessment, Institute of Food Sciences, Warsaw University of Life Sciences-SGGW, 02-787 Warsaw, Poland; tomasz_florowski@sggw.edu.pl; 2 Department of Food Science and Technology, The Ohio State University, Columbus, OH 43210, USA; campanella.20@osu.edu

Recently gels have gained significant attention in the food industry due to their unique properties and potential applications. Among the latest trends in the use of food gels, structural gels should be mentioned. Food gels can be engineered to have specific structures and textures, mimicking the properties of meat, dairy, and fat, making them excellent fat substitutes in products. Further, food gels are being used as carriers for encapsulating and delivering bioactive compounds such as vitamins, antioxidants, and probiotics. These hydrogel-based delivery systems help protect sensitive compounds during processing and storage, and they enable controlled release in the desired target area, such as the gastrointestinal tract. Another innovative way to use food gels is gel films and coatings that are being explored as edible packaging materials that can improve the shelf life of food products, prevent moisture loss, and enhance sensory attributes. These films can be tailored to provide specific barrier properties, such as controlling gas permeability or preventing microbial contamination. There is also an increasing interest in developing food gels with sensing capabilities for monitoring food quality and safety. These smart gels can detect changes in temperature, pH, moisture, or the presence of specific chemicals, enabling real-time monitoring and ensuring product freshness. Advances in 3D printing technology have opened up new possibilities for creating complex food structures using gels. This approach allows precise control over food products′ composition, texture, and appearance, enabling customization and personalized nutrition. That is why current food gels, especially new kinds of gels similar to hydro-[1], aero-[2], and oleogels [3] or emulsion gels [4,5], are essential tools in food product development. They are solid-like structures composed of a liquid phase trapped within a three-dimensional network of biopolymers. Semi-solid colloidal or polymer network food gels have various properties that depend on the polymers used, interactions between their components, mechanisms, or conditions of gelation. By adequately creating the properties of hydrogels, it is possible to have control over syneresis, optimal texture, and enhancing sensory attributes for obtaining innovative food formulations and improved food product quality.

This Special Issue covers different aspects of gel formation [4,6,7,8], properties, including rheological [2,3,4,7,9,10,11], mechanical [4,5,7], and acoustic properties [4], as well as their effect on nutritional properties [3,6] and stability during storage [4,8].

The articles collected in this Special Issue concern creating new, innovative products, the components of which are gel structures. In the research of Jakubczyk et al. [4] on emulsion gels, the effect of mixing rate and the addition of lecithin on the rheological and mechanical properties of agar gels with canola oil was investigated. The results show that higher mixing rates increase the elastic component and hardness of the gels. However, adding lecithin leads to weak gels with low hardness and stability during storage. A new food texture can also be obtained using aerogels, characterized by vast surface area, porous structure, and low weight. Le Thanh–Blicharz et al. [2] study discusses the effects of chemical modifications of starches (E1422, E1450) on the functional properties of aerogels made from normal and waxy potato starches. The research shows that modified potato starch aerogels exhibit lower bulk density and higher oil-binding capacity than native starch. The modifications also enhance their effectiveness as emulsifiers and stabilizers, particularly in the case of E1450 preparation. In searching for new raw materials for food gels, Kim and Iida [6] compared the nutritional characteristics of Tonka beans obtained by different cooking methods and explored their potential as elder-friendly food. Different cooking methods, such as boiling and roasting, are compared, and it is found that roasted Tonka beans have minor nutrient losses and are suitable for elder-friendly food applications. Additionally, the study identifies appropriate concentrations of gelling agents for achieving the desired viscosity standards. Troncoso Recio et al. conducted research on the effect of additives modifying the amino acid composition and properties of wheat dough [9]. The authors investigated the influence of casein hydrolysates and yeast on the rheological properties of wheat dough. Adding casein hydrolysates results in softer gel networks and improved stability, making them effective stabilizing agents. Yeast in the dough creates a more shear-sensitive structure and promotes gel-to-sol transitions. The modification of the properties of the food gels can be carried out by adding more than one polymer to the gel network. Such research was carried out by Adamczyk et al. [7], who studied the rheological behavior of potato starches’ pastes and gels with whole and ground chia seeds. The addition of chia seeds affects the pasting properties of potato starch, and the resulting gels demonstrate new textural properties. Normal starch gels with chia seeds exhibit minor changes in hardness during storage, while waxy starch gels show the opposite effect. Searching also for new starchy raw materials, Wolde et al. [10] focused on the physicochemical, morphological, thermal, and rheological properties of native starches isolated from four cultivars of anchote tubers. Anchote starches show promising functional properties, such as higher gelatinization temperature and enthalpy, improved stability to heating and shearing, and higher viscoelastic moduli compared to potato and cassava starches. These properties make anchote starches suitable for various food product development applications. In addition to new raw materials, one of the options for obtaining food gels with new properties is enzymatic modification. Gharibzahedi and Altintas [8] explored the development of non-fat yogurt gels enriched with stable tarragon essential oil-nanoemulsions (TEO-NEs) using crosslinking of microbial transglutaminase (MTGase). Optimization of the formulation using response surface methodology resulted in the combination of 0.87% TEO-NE and 0.70 U/g MTGase, which achieved desired pH, acidity, reduced syneresis, and increased viscosity and firmness in the yogurt gel. Scanning electron microscopy images showed that the MTGase-induced crosslinks improved the gel structure, enhancing firmness and viscosity while reducing the syneresis rate. The optimal yogurt gel maintained its quality during storage, displaying minimal mold/yeast growth and free-radical oxidation changes. High-protein foods are currently very popular among consumers, especially those with special nutritional needs, and this trend is reflected in Ang et al.’s [11] research, which focuses on the manipulation of mechanical and microstructural properties of whey protein gels using de-structured starch and salts. The study shows that altering ionic strength leads to the formation of distinctive gel structures, which can be utilized in formulating high-protein foods for dysphagia. In creating new foods, substituting ingredients of animal origin with plant-based alternatives is also a trend. Kim et al. [5] developed β-cyclodextrin/konjac-based emulsion gel for a pork backfat substitute in emulsion-type sausage. The addition of β-cyclodextrin enhances the gel strength and improves the gel′s rheological properties and thermal stability. Authors have found the gel formula a suitable fat substitute for low-fat emulsion-type sausages. Finally, the properties of gels, based on ingredients arousing great popularity among consumers, could not be omitted in this Special Issue. The research of Kim and Oh [3] studies the characteristic of insect (Tenebrio molitor larvae) oil (TM oil) for a component of oleogels. Oleogels were prepared using TM oil and different oleogelators, and their rheological and thermal properties were evaluated. The oleogel with carnauba wax exhibited the highest viscoelasticity above 50 °C and had the highest melting point, while the oleogel with candelilla wax had the highest hardness. The carnauba wax oleogel showed promising results as a replacement for shortening in cookies, providing desirable spreadability and texture properties. The last article is a comprehensive review of the research on binary hydrogels [1]. Hilal et al. [1] reviewed the available information on the binary hydrogels′ characteristics, composition, and potential application. This article also presents a bibliometric analysis of research trends in food protein-polysaccharide hydrogels over the past decade. It explores recent developments in hydrogel induction methods and their use as carriers for delivering bioactive compounds in food matrices. The article also emphasizes the potential of using plant-based proteins and polysaccharides to develop food matrices that protect nutrients during processing, storage, and digestion until they reach the targeted area of the digestive system.

This Special Issue provides insights into formulating and optimizing gels for food applications and explores their potential as functional ingredients in various food products.

Finally, we would like to express our sincere gratitude to all the contributing authors who have made significant contributions to the publication of this Special Issue.
